# Circulating microRNA profiles are associated with acute pain and stress in castrated and tail docked lambs

**DOI:** 10.1016/j.vas.2025.100445

**Published:** 2025-03-26

**Authors:** Ryan J. Farr, Christopher Cowled, Carlos Rodrigues, Christina L. Rootes, Dana L.M. Campbell, Caroline Lee, Cameron R. Stewart, Danila Marini

**Affiliations:** a CSIRO Health and Biosecurity, Australian Centre for Disease Preparedness, Geelong, 3220, Victoria, Australia; b CSIRO Agriculture and Food, Armidale, NSW, 2350, Australia; c Adjunct, School of Environmental and Rural Science, University of New England, Armidale, NSW, Australia

**Keywords:** Animal welfare, Biomarkers, microRNAs, miRNAs, Sheep, Stress

## Abstract

Maintaining animal welfare is an essential component of animal production systems. However, multiple measurements are required to inform an animal's welfare state as there are currently no universal measurement tools. Novel biomarkers are increasingly being explored as measures of stress, pain and disease status in livestock. Here we investigate host-encoded microRNAs (miRNAs) as biomarkers of stress and pain to determine the welfare of Australian Merino sheep (*Ovis aries*) following castration and tail-docking. This study used samples from a retrospective trial that compared the effectiveness of pain-relief given to lambs following castration and tail-docking. Plasma collected from lambs at 0 h and 30 mins that received no pain relief post treatment were investigated. Over 1100 novel miRNAs were identified from deep sequencing of small RNA isolated from serum samples. Altered expression of 18 miRNAs was observed in lambs post-castration and tail-docking, of which one miRNA (oar-miR-1–3p) was previously characterised. A supervised machine learning model identified a five-miRNA signature that classified post-castrated and tail-docked sheep from pre-treatment with 99% accuracy. This study describes the characterisation of circulating miRNAs in Merino sheep and demonstrates that miRNAs may have utility for objective measures of animal welfare status in relation to pain.

## Highlights

• Over 1100 novel miRNAs identified from deep sequencing of serum-isolated small RNA.
• Altered expression of 18 miRNAs were observed in castrated and tail-docked lambs.
• Machine learning identified a five-miRNA signature classifying post-procedure lambs. Host-encoded miRNAs are potential biomarkers of stress and pain in sheep.

## 1 Introduction

Determining an animal's welfare state often focuses on the absence of negative states such as pain, stress or disease. Existing approaches to determine welfare state include behavioural assessments and physiological measures, including cardiac function, inflammatory markers and measurements of hormones, such as cortisol. A combination of behavioural and physiological assessment is the most common approach to identifying negative states such as pain and stress in livestock. Most of these measures face significant limitations, including their qualitative nature, reliability, or applicability at the single animal level. One measure alone does not identify welfare differences, and multiple measures are needed to inform welfare state (Barrell, 2019; Tilbrook & Ralph, 2017). For example, assessment of lambs in pain uses a specific ethogram consisting of “abnormal” behaviours typically observed following a painful procedure (Grant, 2004; Stafford, 2013). However, the behaviours observed often depend on the type of pain being experienced (inflammatory, pathological and nociceptive) as well as whether the pain experience is acute or chronic (Marini et al., 2021). In some cases, animals may not display pain related behaviours due to the nature of the species but that does not infer the lack of pain experience (Gougoulis et al., 2010; Johnston et al., 2022; Young, 2006).

A common measure of stress and pain is cortisol, a hormone that is secreted following activation of the hypothalamus-pituitary-adrenal (HPA) axis. Activation of the HPA axis can occur as a result of experiencing pain (Desborough, 2000) and is often used to quantify pain in lambs through increased cortisol concentrations in blood (Marini et al., 2017; Mellor et al., 2002). However, other aspects of production, for example husbandry procedures involving capture and restraint lead to elevated concentrations (Kearton et al., 2019; Turner et al., 2010). Cortisol can also be influenced by positive experiences, with concentrations known to increase with play behaviour (Chapagain et al., 2014) and is influenced by factors such as circadian rhythm (Rietema et al., 2015). It is therefore common to combine behaviour and physiological measures to determine welfare status. Requiring multiple measures can make welfare assessments difficult and time consuming and there is a need for more direct objective measures.

Assessment of animal welfare is constantly improving as new technologies and methods become available. Identification of novel biomarkers, such as microRNAs (miRNAs) is gaining interest as a tool for assessing animal welfare in livestock, proving to be highly specific and accurate as a measure for a variety of welfare states (Marco-Ramell et al., 2018; Miretti et al., 2020; Neethirajan, 2022). Expression levels of miRNAs have been used to identify pain in rodents (Qureshi et al., 2016), horses (Dalla Costa et al., 2021; Lecchi et al., 2018) and pigs (Lecchi et al., 2020) as well as being used to determine other states such as disease and inflammation in cattle (Chen et al., 2014; Li et al., 2014; Tzelos et al., 2022) and heat stress in sheep (Lu et al., 2019).

MicroRNAs are single-stranded non-coding RNAs that mediate post-transcriptional regulation of protein-coding genes. MicroRNAs typically function as a guide by base-pairing with target miRNAs to reduce gene expression via the RNA-induced silencing complex (RISC) (Kawamata et al., 2009). There are currently over 1000 functional mature miRNAs listed in the human genome, with another approximate 1000 predicted (miRBase: Griffiths-Jones, 2006). MicroRNAs collectively regulate approximately 30 % of human protein-coding genes (Lewis et al., 2005) involved in most cellular and physiological events, including stress (Leung & Sharp, 2010).

The aim of this study is to characterise circulating miRNAs in sheep and to perform a proof-of-concept study investigating circulating miRNAs as biomarkers of stress and pain to help determine sheep welfare state objectively. We hypothesize that physiological events associated with stress or pain in sheep would trigger changes in the expression of select circulating miRNAs. Results from this pilot study provide insight into the previously unappreciated role of host miRNAs in the animal stress response and suggest a potential role for miRNAs in animal welfare monitoring.

## 2 Materials and methods

### 2.1 Animal ethics

Samples were obtained from a retrospective study conducted at CSIRO's McMaster Laboratory, Armidale, New South Wales (NSW), Australia. Protocol and conduct were approved by The CSIRO Chiswick Animal Ethics Committee under the NSW Animal Research Act, 1985 (approval ARA 14/21 (Marini et al., 2017)).

### 2.2 Animal study

The study by Marini et al. (2017) investigated the effectiveness of the non-steroidal anti-inflammatory, flunixin, administered orally versus injectable in lambs that underwent castration and tail-docking. The study was a block design, using 64 10-week-old male Merino lambs. Lambs were blocked by weight and randomly allocated within block to one of four treatments. 1) Handled control group; 2) Castration and tail-docking no pain relief; 3) Castration and tail-docking oral flunixin; and 4) Castration and tail-docking injectable flunixin. Blood was collected from the lambs by jugular venepuncture using 21-gauge needles into 4.5 mL vacutainers containing EDTA. Samples were taken at 0 h, 30 min, 6 h, 12 h, 24 h and 48 h, relative to treatment application. All animals in all treatment groups had normal cortisol and haematological variables at baseline (0 h) prior to treatment application. Comparison of control lambs to castrated and tail-docked lambs with and without pain relief revealed that the change in behaviour, increase in cortisol, haptoglobin and presence of inflammatory markers (neutrophil, lymphocyte cells) was a result of the pain of the husbandry procedure itself, and not handling. Comparison of lambs receiving pain-relief to those without, showed that pain-relief reduced cortisol and inflammatory markers. Blood was analysed for cortisol; full details of the experiment are described in Marini et al. (2017). Samples collected at 0 h and 30 min from lambs in the castration and tail-docking with no pain relief group were used. Forty-two samples were included from lambs that had known cortisol increases (n = 10 for pre-castration and tail docking, n = 11 for post-castration and tail-docking).

### 2.3 RNA isolation and RNA-seq

Plasma was collected from EDTA blood samples by centrifugation as previously described (Farr et al., 2021) and randomised for library preparation. Total RNA was isolated from 200 µL of plasma using the miRNeasy micro kit (catalogue number ID. 217,084) (Qiagen, Hilden, Germany) as per manufacturer's protocol with a modification: after lysis with Qiazol (catalogue number ID. 79,306), 10 µg of glycogen (Sigma Aldrich, St. Louis, USA, G1767) was added to each sample to as a carrier of miRNA. Complementary DNA libraries were prepared using the QIASeq miRNA Library kit (catalogue number ID. 331,502), and QIASeq miRNA NGS 48 Index IL (catalogue number ID. 331,565), (Qiagen) as per the manufacturer's protocol (handbook HB-2157–009 April 2021) with a modification: libraries underwent 24 cycles of amplification. Libraries were analysed using the Bioanalyser 2100 on a High Sensitivity DNA kit (catalogue code 5067–4626) (Agilent, Santa Clara, USA) to confirm correct insert size and minimal adapter or primer carryover. Libraries were sent to the Australian Genome Research Facility (AGRF) for 100 bp and sequencing on the NovaSeq 6000 (Illumina, San Diego, USA). Small RNA sequencing resulting in 25–55 million raw reads per sample. Reads were trimmed to 18–26 nucleotides using CutAdapt (Martin, 2011). Quality was assessed using FastQC (www.bioinformatics.babraham.ac.uk/projects/fastqc/). Filtering on length and quality resulted in a loss of 70–90 % of raw reads, leaving 0.2–3 million reads per sample. MiRNAs with at least 1 count per million in three or more samples were analysed further.

### 2.4 miRNA expression analysis

MiRDeep2 (Friedländer et al., 2012) was used to identify miRNA transcripts, with total counts including all reads that mapped to a locus (as opposed to reads matching the canonical/consensus sequence only) against the miRBase bovine miRNA database. Read normalisation was performed using DESeq2 (Love et al., 2014) in R (Version 4.3.1) via the *DESeq()* function, which estimates size factors via the median-of-ratios method to correct for differences in sequencing depth across samples. These size factors are then applied to the raw counts, ensuring that technical variability due to size is minimised, allowing for accurate comparison of gene expression levels between groups.

### 2.5 Characterisation of novel miRNAs in merino sheep (*Ovis aries*)

MiRNAs were identified in the small RNA-Seq data using miRDeep2 (Friedländer et al., 2012). Briefly, reads were checked for quality using FastQC (Andrews, 2010), then trimmed using Cutadapt (Martin, 2011) to remove the QIAseq miRNA NGS 3′ Adapter (AACTGTAGGCACCATCAAT). Short and long reads were discarded to retain sequences in the range of 18–26 nucleotides. Reads were then converted to FASTA format using the FASTX toolkit (Hannon, 2010), and all samples were concatenated into a single file. Reads were then mapped to the sheep genome (Oar_v3.1, Ensembl) using Bowtie2 (Langmead B & S., 2012) via the mapper.pl script provided by miRDeep2. To run miRDeep2, the *reference this species* fields were provided with the set of all known sheep miRNAs from the latest release of miRBase (currently version 22) (Griffiths-Jones, 2006). For the *reference other species* field, we used the set of miRBase22 horse miRNAs. A minimum miRDeep2 score cutoff of ≥ 1.0 was applied to selection of novel candidates, while all known miRNAs were retained regardless of score. To construct the Venn diagram, we used the set of all known mature miRNA sequences from vertebrates other than sheep, also obtained from miRBase22.

### 2.6 Machine learning classifier

Five machine learning (ML) classification models (Support Vector Classifier, Logistic Regression, Decision Tree Classifier, Stochastic Gradient Descent Classifier and Random Forest were assessed. Analyses were conducted using the scikit-learn module in Python (Pedregosa et al., 2011). Highly correlated miRNAs (Pearson correlation >0.8 or <−0.8) had one member removed to avoid introducing skewed or misleading results (Farr et al., 2021). Subsets of miRNAs were selected based on a forward greedy stepwise selector and the recursive feature elimination (Guyon et al., 2002) approach to determine the minimum number of miRNAs needed to accurately categorise groups. Each model underwent hyperparameter tuning using the GridSearchCV functionality, and model performance was assessed by randomly splitting the data into 80 % labelled training data and 20 % unlabelled testing data. This process was repeated 100 times to determine average model performance using different random splits. Accuracy (proportion of correctly classified instances), precision (proportion of true positive predictions among all positive predictions), recall (proportion of true positive predictions among all actual positive instances), F1 (harmonic mean of precision and recall) and Receiver Operative Characteristic Area Under the Curve score (ROC AUC; a metric which considers both true and false positivity rates) scores were determined for each model.

## 3 Results

**Fig. 1:**
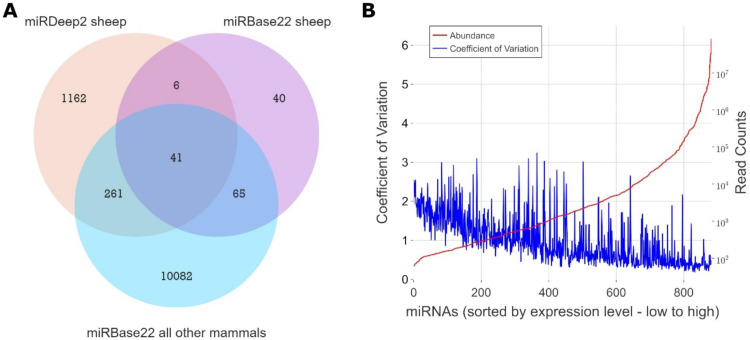
Overview of miRNA expression in sheep sera (A) Comparison between sheep miRNAs sequenced in this study (miRDeep2) and reference miRNAs from miRBase22 (miRBase22 sheep, miRBase22 all other mammals). (B) Inter-sample variance (blue line) decreases with increasing depth of sequencing (red line).

Reads originating from 836 distinct miRNA precursors were detected, of which 715 were not previously described in sheep. From this, 1470 distinct mature miRNA sequences were predicted, of which 1341 were directly observed in the sequencing data. 5p/3p suffixes were added to mature miRNA labels including known ones, e.g.oar-mir-16b-5p, while novel candidates were assigned temporary labels, e.g.oar-temp-685–3p. In total, 308 sheep mature miRNAs matched with 100 % identity to known sheep or other vertebrate mature miRNAs in miRBase22 (Fig. 1A). Many more featured high similarity to known entries but were not identical (not shown). To examine the relationship between abundance and inter-sample variance, miRNAs were sorted by increasing read depth and plotted on a log_10_ scale against their coefficient of variation (CoV) (Fig. 1B). The CoV graph revealed a relatively clear relationship between read depth and variance, with more abundant miRNAs showing lower levels of variation in expression levels than less abundant miRNAs.

**Fig. 2:**
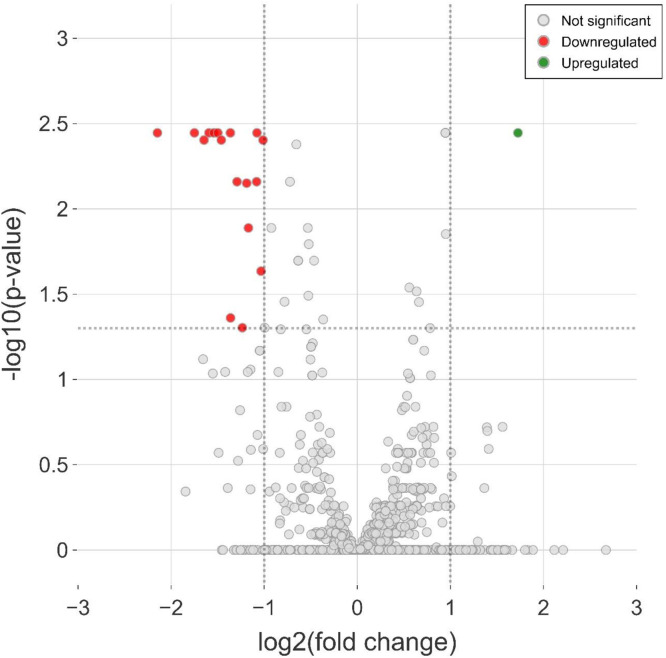
Tail docking and castration induce miRNA changes in sera. Volcano plot showing the increased (fold change greater than 1) and decreased (fold change below −1) differentially expressed (DE) miRNAs in post tail dock and castration cases compared to pre procedure. Horizontal dotted line marks the p-value cut-off (False Discovery Rate, FDR<0.05) and vertical lines are the fold change cut-off (>1 FC). miRNAs above the dotted line between a fold change of −1 and 1 are statistically significant but are not >1 FC.

Eighteen miRNAs were differentially expressed in sheep when comparing samples from before and after castration and tail docking. This dataset consisted of a single up-regulated miRNA in tail docked and castrated animals (oar-temp-7–5p) and 17 down-regulated miRNAs (Fig. 2, Table 1). An additional 19 miRNAs were differentially expressed with log_2_FC values <1. The most down-regulated were oar-temp-214–3p (−2.15 log_2_FC), oar-temp-423–3p (−1.75 log_2_FC) and oar-temp-214–3p (−1.54 log_2_FC).

**Table 1:** Host-encoded miRNAs differentially expressed following tail-docking and castration.

miRNA	baseMean	log2FoldChange	padj
oar-mir-1–3p	1381.88	−1.59	0.003583062
oar-temp-118–5p	26.29*	−1.65	0.003948117
oar-temp-126–3p	5.32*	−2.15	0.003583062
oar-temp-214–3p	228.14	−1.54	0.003583062
oar-temp-25–3p	844.45	−1.08	0.003583062
oar-temp-29–3p	1254.17	−1.19	0.007066883
oar-temp-377–5p	11.41*	−1.23	0.049746643
oar-temp-423–3p	612.41	−1.75	0.003583062
oar-temp-46–3p	365.48	−1.50	0.003583062
oar-temp-47–3p	369.83	−1.46	0.003948117
oar-temp-50–3p	233.35	−1.36	0.003583062
oar-temp-6–3p	6799.52	−1.01	0.003948117
oar-temp-6–5p	2065.57	−1.08	0.006927485
oar-temp-68–3p	37.42*	−1.29	0.006927485
oar-temp-68–5p	35.20*	−1.36	0.043579942
oar-temp-7–5p	10,442.35	1.73	0.003583062
oar-temp-79–5p	67.65	−1.17	0.012934198
oar-temp-83–3p	48.02	−1.03	0.023173217

**Fig. 3:**
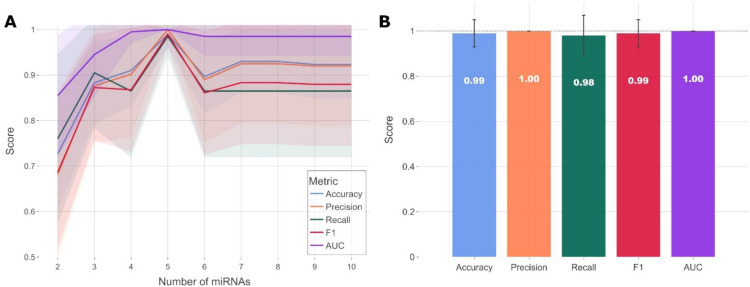
A miRNA signature in sera classifies tail docking and castration with >95 % accuracy. (A) Feature (miRNA) selection line plot showing the impact of increasing numbers of miRNAs on the performance of a logistic regression model. miRNAs were selected using the recursive feature elimination approach. Each combination of miRNAs was randomly assessed 100 times. Shaded areas are the 95 % CI. (B) Bar plot showing the mean score of the miRNA signature in predicting castration and tail docking. Error bars are the 95 % CI after 100 random iterative assessments.

We next investigated if the circulating miRNA profile could independently classify tail docking and castration. A supervised machine learning method was implemented for the identification of the most predictive miRNAs and refined to identify the minimum targets necessary for prediction and classification. The most predictive miRNAs were selected using recursive feature elimination (Fig. 3A) in a model trained using the Logistic Regression algorithm. Measuring five miRNA targets (oar-miR-1–3p, oar-miR-10a-5p, oar-miR-23b-3p, oar-miR-27a-3p and oar-temp-5–5p) in combination resulted in a model with an average performance of 99 % accuracy, 100 % precision and 98 % recall, with a ROC AUC of 1 (Fig. 3B, Table 2). Increasing candidates within the biomarker signature to more than five miRNAs did not improve classification performance.

**Table 2:** Host-encoded miRNAs predictive of tail-docking and castration classification.

miRNA	baseMean	log2FoldChange	padj
oar-miR-1–3p	1381.88	−1.59	0.003583
oar-miR-10a-5p	1283.78	−0.46	0.020117
oar-miR-23b-3p	29,947.24	−0.37	0.044491
oar-miR-27a-3p	525.38	−0.92	0.012934
oas-temp-5–5p	14,772.82	0.64	0.03044

**Fig. 4:**
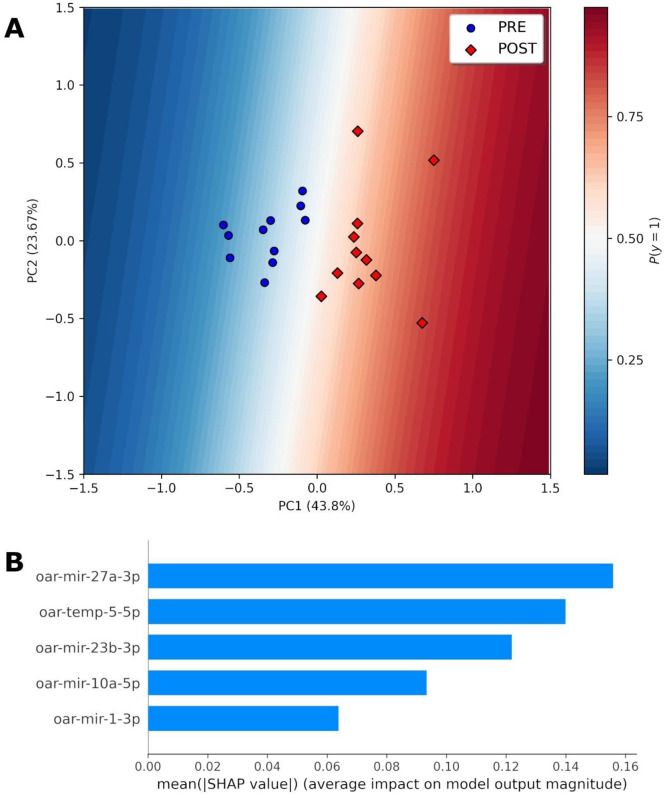
Decision boundary visualisation for a binary classification model using principal component analysis. The original dataset with five-miRNA biomarker was reduced to two dimensions using PCA to enable 2D visualisation. The plot illustrates the classifier's decision boundary, where the background colour gradient represents the model's prediction probability from low (blue) to high probabilities (red). Data points are overlaid as circles and diamonds, representing samples of the pre and post castration/tail dock groups, respectively. (B) A SHAP bar plot demonstrating the impact of each miRNA feature, in order of importance based on mean absolute SHAP values, on the tail docking/castration prediction.

A decision boundary graph, based on the first two principal components (PC1 and PC2) of the original dataset with the five-miRNA biomarker signature, showed clear distinctions between animals that were castrated, and tail docked before and after the procedure (Fig. 4A). The decision boundary graph also clearly shows that each sample's grouping was predicted with a high degree of confidence. MiRNAs oar-miR-27a-3p, oar-temp-5–5p and oar-miR-23b-3p had the highest impact on the model in descending order of importance, with Shapley values ranging from 0.12 to 0.16 (Fig. 4B).

## 4 Discussion

There is a growing appreciation of miRNAs as biomarkers for livestock health and welfare. Indeed, ruminant miRNA responses have been measured in response to stressors including infection, inflammation, weaning (Guyon et al., 2002), changes to social structures hierarchy (Colitti et al., 2019), heat stress (Li et al., 2018; Zheng et al., 2014), and hypoxia (ZhiWei et al., 2019). Efforts to develop welfare biomarkers in livestock can be hampered by poorly annotated miRNA reference lists. Having previously characterised known and novel miRNAs in horse sera, we have performed a comparable analysis to considerably expand the sheep miRNA reference list. Our analysis identified 1162 novel miRNAs not previously observed in the miRBase22 sheep reference, which contained 152 candidates. Expanding the catalogue of known miRNAs in ruminant biofluids aids in efforts to progress the biomarker discovery process.

Current methods of measuring pain in animals in both a production and laboratory setting can be subjective and hard to determine (Zentrich et al., 2023). This leads to the implementation of multiple measures (behavioural, physiological and immunological) to infer the level of pain the animal may be experiencing. With the concern for animal welfare only continuing to increase (Cornish et al., 2019), it is important we are able to accurately identify animals in a negative welfare state. The general public consider invasive husbandry procedures such as castration and tail docking as welfare issues (Phillips et al., 2009) with the presence of pain and fear being important welfare indicators (Doughty et al., 2017). In Australia, community attitudes towards controversial practices within the livestock industry have led to negative perceptions of those livestock industries (e.g. mulesing and live export ban) (Coleman, 2018). Having the ability to accurately identify sheep in pain leads to increased provision of pain relief to those animals (Crawford et al. 2023), which improves animal welfare and can help maintain the social licence to farm.

The plasma samples used were from Marini et al., 2017 and were collected from animals at 0 h (prior to castration and tail docking) and 30 mins after castration and tail docking. The lambs in this group were not provided with pain relief at the time of treatment. Using standard measures of pain for lambs (behaviour, cortisol and inflammation) indicated that 30 min was the peak of the acute pain experience in these animals. It is also known that the response of these lambs was directly in response to pain and not to the stress of handling as sham control animals in the study (those only handled in a similar manner with no treatment implemented) experienced no changes to cortisol, behaviour or inflammation in the same period post treatment. This current study found several unique markers that were expressed in the lambs only after the pain of castration and tail docking had been experienced. Through miRNA expression analysis and characterisation of novel miRNAs in Merino sheep we were able to identify eighteen miRNAs that were differentially expressed in sheep plasma before and after castration and tail docking. Of these, 5 were able to independently classify tail docking and castration. Having objective miRNA measures that can indicate the presence of pain can give us a clear yes or no as to whether an animal is in pain. This could have implications for accurately identifying animals experiencing pain leading to individual targeted treatment. In pigs, use of miRNAs as pain biomarkers were able to identify individuals that did not receive analgesia (Lecchi et al., 2020). Thereby, use of miRNA expression may be an additional measure that can determine the efficacy of analgesics for painful husbandry practices.

As new technologies emerge to objectively measure animal welfare and stress, so too comes the requirement to deliver practicable and feasible solutions. Presently, miRNAs are commonly measured using laboratory-based qRT-PCR assays. Similarly, the requirement to draw blood and prepare sera also lends itself to a laboratory-based assay. Understanding the application, context and end-use of new technologies is a critical driver towards adoption. For instance, laboratory-based assays are compatible with high-throughput testing and may offer advantages in test accuracy, reliability and standardisation. On the contrary, point-of-care tests may enable faster results, lower loss and greater end-user ownership. Adoption and acceptability would also require regulatory approvals beyond pilot studies such as the present study.

Future work will expand upon initial findings made in this study, including validating miRNA responses in larger cohorts of sheep subjected to tail docking and castration, and comparing responses to other stress and welfare models, including physical (e.g. dehorning, bruising, infighting) and psychological (e.g. weaning, novel environments, co-mingling, travel) stressors. Validation studies may also include assessments of biomarker robustness (i.e. a comparison of results when tests are performed by different operators on different days using different laboratory supplies). As miRNAs are often highly conserved between species, it will be intriguing to measure the level of conservation of stress-related miRNA responses in ruminants. Such analyses will help determine the applicability of miRNAs as widely used stress biomarkers, compared to stress hormones such as corticosterone. Similarly, characterising the cellular origin of miRNAs observed in this study will categorise miRNA responses to stress versus other aspects of the tail docking and castration process, such as wound healing. The identification of miRNAs regulating activity of hypothalamic-pituitary-adrenal (HPA) axis, will prove crucial in this regard.

## Animal ethics approval

Samples were obtained from a retrospective study conducted at CSIRO's McMaster Laboratory, Armidale, New South Wales (NSW), Australia. Protocol and conduct were approved by The CSIRO Chiswick Animal Ethics Committee under the NSW Animal Research Act, 1985 (approval ARA 14/21 (Marini et al., 2017)).

## Declaration of generative AI and AI-assisted technologies in the writing process

The authors did not use any artificial intelligence assisted technologies in the writing process.

## Financial support statement

This work was supported by the Commonwealth Scientific and Industrial Research Organisation (CSIRO, Canberra, ACT, Australia).

## CRediT authorship contribution statement

**Ryan J. Farr:** Writing – review & editing, Writing – original draft, Visualization, Methodology, Investigation, Funding acquisition, Formal analysis, Data curation. **Christopher Cowled:** Writing – original draft, Visualization, Methodology, Investigation. **Carlos Rodrigues:** Writing – original draft, Visualization, Validation, Methodology, Investigation, Formal analysis, Data curation, Conceptualization. **Christina L. Rootes:** Validation, Investigation, Formal analysis. **Dana L.M. Campbell:** Writing – review & editing, Writing – original draft, Methodology, Investigation, Conceptualization. **Caroline Lee:** Writing – review & editing, Writing – original draft, Resources, Funding acquisition, Conceptualization. **Cameron R. Stewart:** Writing – review & editing, Writing – original draft, Visualization, Methodology, Investigation, Funding acquisition, Formal analysis, Data curation. **Danila Marini:** Writing – review & editing, Writing – original draft, Resources, Methodology, Investigation, Conceptualization.

## Declaration of competing interest

The authors declare that they have no known competing financial interests or personal relationships that could have appeared to influence the work reported in this paper.

## Data Availability

None of the data were deposited in an official repository.
